# Transgenerational consequences of PTSD: risk factors for the mental health of children whose mothers have been exposed to the Rwandan genocide

**DOI:** 10.1186/1752-4458-8-12

**Published:** 2014-04-01

**Authors:** Maria Roth, Frank Neuner, Thomas Elbert

**Affiliations:** 1 Department of Psychology, University of Konstanz, 78457 Konstanz, Germany; 2 Clinical Psychology and Psychotherapy, Bielefeld University, Postbox 100131, 33501 Bielefeld, Germany

## Abstract

**Background:**

Understanding how parental Posttraumatic Stress Disorder (PTSD) may or may not affect the development and mental health in the offspring is particularly important in conflict regions, where trauma-related illness is endemic. In Rwanda, organised atrocities and the genocide against the Tutsi of 1994 have left a significant fraction of the population with chronic PTSD. The aim of the present investigation was to establish whether PTSD in mothers is associated with symptoms of depression, anxiety, and aggressive and antisocial behaviour in their children.

**Methods:**

A community sample of 125 Rwandan mothers who experienced the genocide of 1994 and their 12-year-old children were interviewed. Using a structured interview, symptoms of maternal PTSD and children’s depression, anxiety, and aggressive and antisocial behaviour were assessed by trained and on-site supervised local B.A. psychologists. The interview also included a detailed checklist of event types related to family violence.

**Results:**

In showing that a maternal PTSD was not associated with child’s psychopathology, the results contradict the assumption of straight “trans-generational trauma transmission”. Instead, a child’s exposure to maternal family violence posed a significant risk factor for a negative mental health outcome. Furthermore, it was not maternal PTSD-symptoms but mother’s exposure to family violence during her own childhood that was associated with the magnitude of adversities that a child experiences at home.

**Conclusions:**

Contrary to a simple model of a trans-generational transmission of trauma, neither maternal PTSD nor maternal traumatic experiences were directly associated with symptoms of anxiety, depression, or antisocial and aggressive behaviour in the children. Instead, the present results suggest a relationship between parental child rearing practices and children’s mental health. Furthermore, the study details the “cycle of violence”, showing a significant link between maternal violence against a child and its mother’s experience of childhood maltreatment.

## Background

An association between parental exposure to traumatic stress and children’s psychological mental health has been suggested by studies that examined families of veterans. Children of fathers suffering from PTSD showed significantly greater internalized and externalized behavioural problems [1,2], somatic complaints [3] and higher scores of depression [3,4] and anxiety [5] compared to children of veterans who did not present with a mental disorder. An increased level of behavioural disorders, anxiety and depressive symptoms, as well as posttraumatic stress were also confirmed in offspring of tortured refugee parents suffering from PTSD compared to children with non-traumatized parents [6,7]. Although children whose mothers and fathers both had PTSD show significantly higher scores of psychopathology such as anxiety and depression, the mother’s anxiety was identified as the most frequent and important predictor of children’s mental health status [4].

While systematic studies assessing the association of children’s mental health status and parental traumatization are rare, a number of examinations focused on changes and impairments in the family system that stem from parental PTSD and trauma exposure. Several studies have found general impairments of child rearing capacities, such as inadequate emotional reaction [8], impaired parent–child relationship [9,10], disrupted communication styles [11] or physical punishment [12-15]. Physical punishment was often explained by hyperarousal symptoms or substance abuse in the parents [16] and was associated with combat experiences in veteran fathers and behavioural problems in the offspring [17]. Similar to the *dose effect* for vulnerability to PTSD [18,19], a dose effect for the consequences of family violence was found: children who experienced more maltreatment in childhood demonstrated more severe behavioural problems and higher rates of delinquency [20,21].

The possibility of trans-generational consequences of parental PTSD arises specifically in populations with a high prevalence of PTSD. Rwanda represents a nation with a history of numerous massacres, most devastating the genocide from 1994, killing 10% of Rwanda’s almost 8 million inhabitants, mostly Tutsi and oppositional Hutu [22]. Consequently, even more than a decade after this massive violent event, the prevalence of PTSD has remained exceptionally high in Rwanda [23-26]. Additionally, the Rwandan population has to cope with experiences of massive bereavement and extensive social disruption [27]. Furthermore, Rwandans still remain in a state of hyper-vigilance and trepidation, worrying that genocidal hostilities between the two ethnic groups might occur again [23]. We suppose that existing parental psychopathology resulting from past traumatic experiences and current worries about reoccurrence of the slaughters have a substantial psychological impact on the mental health of the offspring generation. Therefore, the examination of trans-generational consequences of parental PTSD in Rwanda may help to better understand the long-term impact of parental PTSD for children’s mental health, and to detect individual, familial and social needs in order to support a psychological healing process in Rwandan society.

**Figure 1:**
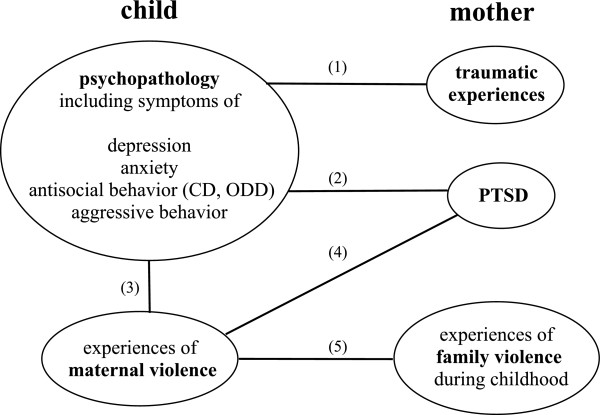
Diagram illustrating the causal pathways of the tested hypothesis.

The goal of the present study was to investigate the consequences of maternal traumatization for their children in a non-clinical setting. There is an on-going debate about *trans-generational trauma transmission* due to methodological flaws in studies investigating this phenomenon, such as the use of non-random samples, the lack of a control group, small sample sizes, unclear definition of “traumatized parent” or the use of non-valid instruments for assessment [28,29]. In this study, we defined a mother as being “traumatized” when she fulfilled the DSM-IV diagnosis of PTSD. This definition is one possibility for specification of the psychological state in the maternal generation and will allow comparison of the mental health of children according to the PTSD diagnosis of the mother. We aimed to examine the effects of maternal PTSD on the mental health of their school-aged children (born two years after the genocide) in the Rwandan context, including symptoms of depression, anxiety, and aggressive and antisocial behaviour. We focused our assessment on the mothers and not the fathers as studies support an increased impact of maternal anxiety on the children’s mental health status compared to the father’s mental wellbeing. In line with previous theory on transmission mechanisms we assumed that family violence constitutes an important impact on children’s mental health. We predicted that (1) children’s psychopathology is associated with the number of maternal traumatic experiences; (2) children’s psychopathology is associated with maternal PTSD; (3) children’s psychopathology is associated with the experiences of maternal violence at home; (4) children’s experiences of maternal violence at home are associated with maternal PTSD; and (5) children’s experiences of maternal violence at home are associated with maternal experiences of family violence during her childhood. See Figure 1 for a diagram, which visualizes our hypothesis.

## Methods

### Sample

From April to May 2008 we conducted a community-based survey in Rwanda in order to examine trans-generational consequences of PTSD in mothers who experienced the genocide in 1994 and their 12-year-old children who were born two years after the genocide.

The recruitment took place in three different public schools in Butare in southern Rwanda. In order to achieve a wide range of socio-economic status in the examined families, recruitment took place at two rural, and one urban schools. These schools were chosen according to logistical aspects. Participants were recruited according to the following criteria: (1) children were 12 years old, (2) children lived together with the biological mother, and (3) the mothers had lived in Rwanda during the time of the genocide in 1994. We have focused on biological mothers in order to exclude additional confounding variables, which may derive from the experienced stress in the children with the change of the most important caretaker. Approval for the survey was obtained from the National Institute of Statistics of Rwanda and the Ethical Review Board of the University of Konstanz, Germany.

#### *Sample characteristics*

A convenient sample of 125 women and their 12-year old children (41 boys and 84 girls) were interviewed. The mothers were on average 42 years old (range = 24 – 59), 52% (n = 65) were married, 36% (n = 45) were widows, 10% (n = 13) divorced or separated, and 3 mothers (2%) were single. The children reported having on average 3 to 4 siblings (mean = 3.87; range = 0 – 10; SD = 2.04) and attended the second (9%, n = 11), third (31%, n = 28), forth (40%, n = 50) and fifth grade (20%, n = 25). The majority of the households owned a house/hut (81%, n = 101), 72% (n = 90) possessed a vegetable garden and 8% (n = 10) grew crops in order to sell them. A global index of the household’s economical status was created in order to test its association with children’s psychopathology. The index was calculated by adding up the z-standardized scores of family possession, such as furniture (table, beds, carpets), books, cooking pots, bikes, motorbikes, television, radio, property (house, garden), farm animals, children’s possession (number of toys and cloths), as well as the family’s average monthly income and the number of meals per day of mothers and children. This indicator ranged from -15 to 27.

### Procedure

The children were selected by the directors and teachers of the schools according to the inclusion criteria. The teachers first informed the children to ask their mother to come to the school on the following day, where they were informed about the investigation by the interviewers. After the mothers have agreed to take part in the study, the interviewers accompanied them to their home, where the interview took place. None of the mothers refused to participate in the study. At home in a confidential setting a more detailed explanation of the study was given. If the mother still agreed to participate, she signed a written informed consent for herself and for her child. Each mother was interviewed at her home and subsequently the child was investigated at the school. Six of the originally-recruited 131 mother-child pairs were excluded since the interviewed caregiver turned out not to be the biological mother of the child. The interviews were conducted by a group of six Rwandan B.A.-level psychologists recruited from the National University of Rwanda in Butare. The interviewers had been previously trained by colleagues from the University of Konstanz in conducting interviews in order to assess PTSD, depression, anxiety and suicidal tendencies [30]. The interviewers attended an additional training for this study. The training lasted eight days and included theoretical sessions on the purpose of the study, concepts of trauma, the questionnaires, and role-plays conducting the survey. The project coordinator closely supervised the interviewers during the whole period of data collection.

### Instruments

All instruments were available in their original English version. A professional translator created a Kinyarwanda language version of all questionnaires. The translated version was back-translated and discussed with the local interviewers in the training and corrected where necessary. All diagnostic instruments were administered as clinical interviews.

#### *Assessment of traumatic experiences and PTSD in mothers*

An extensive list of 25 items adapted to the Rwandan genocide was used to assess traumatic experiences and included items such as “Have you seen dead or mutilated bodies?” or “Were you forced to hide under dead bodies?” [26]. The *Posttraumatic Symptom Scale – Interview Version (PSS-I) *[31] was chosen to assess symptoms of PTSD in the mothers. The severity of a symptom is rated on the basis of its frequency, intensity/severity, or both. The diagnosis for PTSD was created regarding the DSM-IV criteria. The procedure has been validated in East African settings [32,33] and both instruments have been used by our team in the Rwandan context previously. Symptoms of trauma can be observed in any culture [34]. The debate about a nosological entity of PTSD in various cultures is not relevant in this context. “PTSD” in this case simply refers to a set of symptoms that obviously can be observed in any culture.

#### *Assessment of depression, anxiety, antisocial and aggressive behaviour in children*

With the help of a subscale of the *Hopkins Symptom Checklist (HSCL) *[32,35], children‘s anxiety symptoms occurring during the last seven days were assessed. Symptom intensity was rated on a scale from 0 (“not at all”) to 3 (“extreme”). Symptoms of depression were acquired with the 10-item short version of the *Children’s Depression Inventory (CDI-S)*, an instrument appropriate for children aged 7 to 17 years [36]. Each item is composed in three different questions, which represent the different intensity of a symptom and scores from 0 to 2. For example: (0) “I am sad once in a while”; (1) “I am sad many times” and (2) “I am sad all the times”. The summed score of the items ranges from 0 to 20, with a higher score representing greater depressive symptoms. Clinical relevant symptom scores of anxiety and depression were identified according to cut-off recommendations [36,37].

Conduct Disorder (CD) and Oppositional Deviant Disorder (ODD) were assessed with the help of the Sections P and Q of the *M.I.N.I. Kid *[38]. In order to apply the instrument adequately in the Rwandan context, the cut-off for *normal – not normal* for every item was discussed in the training with the local interviewers. Additionally, a short version of the aggression scale by Buss and Perry [39] was used to assess aggressive behaviour in the children. It contains 16 items, with subscales for physical and verbal aggression, anger and hostility. Answers range from 0 (“I don’t agree”) to 4 (“I totally agree”), with a higher score representing higher aggressive behaviour and attitude, with a maximum sum score of 64.

A score of children’s psychopathology was generated by adding up the standardized (z-transformed) symptom sum scores for anxiety, depression, aggression, ODD, and CD. The score ranged from -5.7 to 9.8 (0 ± SD 3.33).

#### *Assessment of family violence in mothers and children*

The *Checklist of Family Violence (CFV) *[40] was used to assess experiences of family violence in mothers and children. The 36 item checklist defines familial violence as exposure to neglect, physical, emotional or sexual violence as well as witnessing violence between other family members. Additional items at the end of the checklist asked about the identification of the perpetrators of the violence (multiple possible answers), and the interviewee’s subjective psychological experience upon experiencing the violence, e.g., “Did you feel helpless?” The participant could answer the questions with “yes” or “no”. In this study we asked the child to identify every event where the mother was the perpetrator of the violent act. The questionnaire had previously been used in other cultural contexts with children [41,42]. With the help of this checklist, the mothers’ previously experienced family violence during childhood and the children’s experienced family violence at home was assessed. For mothers, two items were added to assess intimate partner violence and conjugal rape.

### Statistical analysis

For calculations of Pearson correlations and t-test the following symptom and event sum scores were used: maternal traumatic event types, children’s psychopathology, children’s experienced maternal violence. Group differences were calculated according to maternal PTSD diagnosis, defined according to DSM-IV criteria. In order to explore how different factors contribute to the psychopathology of the children and children’s experienced maternal violence at home, we calculated hierarchical linear regression model. These included potential confounding covariates, such as children’s gender, number of siblings, educational level (current school grade) and the family’s economical status, all of which did not significantly explain any further variance in children’s psychopathology. A priori there was insufficient knowledge to reasonably estimate the effect size, and the usefulness of post-hoc power analyses remains controversial [43]. As Hoenig and Heisey [44] show, all post-hoc power analyses suffer from what is called the "power approach paradox". Given that post-hoc power is a function of the p-value attained, it adds little information. Analyses were performed with SPSS version 21 for Macintosh.

## Results

### Violent experiences and PTSD in mothers

On the *CFV* nearly all mothers (n = 124, 99%) reported having experienced at least one event of familial violence during their childhood, counting 6.9 different event types on average (range = 0 – 18; SD = 4.71). Most of the mothers (n = 117, 94%) had been exposed to physical violence, 59% (n = 74) to emotional violence, 28% (n = 47) had experienced events of neglect, and 86% (n = 107) had witnessed violent acts committed against a family member. The most frequently mentioned items were *being hit with an object* (n = 116, 92%), *having witnessed a family member being hit with an object* (n = 96, 77%), and *being slapped on the body, arms or legs* (n = 64, 51%). More than every other mother (n = 67, 55%) who was married once in her life reported experiences of physical violence by her husband; 27% (n = 33) had been forced into sexual intercourse.

With the help of the adapted event list, the mothers reported an average of 11 different types of traumatic events in their lives (range = 4 – 21, SD = 4.31) of which 7 (range = 1 – 17, SD = 4.28) were associated with the genocide in 1994. The most frequently-reported traumatic event types were *being forced to flee* (n = 119, 95%), *having witnessed an armed attack* (n = 109, 87%), and *needing to hide* (n = 86, 69%). The majority (60%) of the traumatic events, which were described as the worst experience, occurred during the genocide in 1994. According to DSM-IV criteria, every fourth mother (n = 43, 26%) fulfilled the diagnosis for PTSD, assessed with the *PSS-I*.

### Experiences of family violence in children

Questioned with the *CFV*, nearly all children (n = 124, 99%) reported at least one incident of family violence, counting 5.6 different violent events on average (range = 0 – 15, SD = 3.15). Physical violence was experienced by 99% (n = 124), emotional violence by 50% (n = 62), neglect by 22% (n = 27) and 81% (n = 101) witnessed family violence against another family member. The children identified the mother (n = 114, 91%), and older siblings (n = 62, 50%) as perpetrators of family violence. 39% (n = 47) of those children whose mother had ever been married, reported the father as perpetrator of family violence. Most frequently reported items were *being hit with an object* (n = 123, 98%), *having witnessed a family member being hit with an object* (n = 91, 73%), or *slapped on the body, arms or legs* (n = 77, 62%). The majority of the children felt terrified (55%) or helpless (51%) because of family violence.

### Children’s psychopathology in association with maternal traumatic experiences

**Figure 2:**
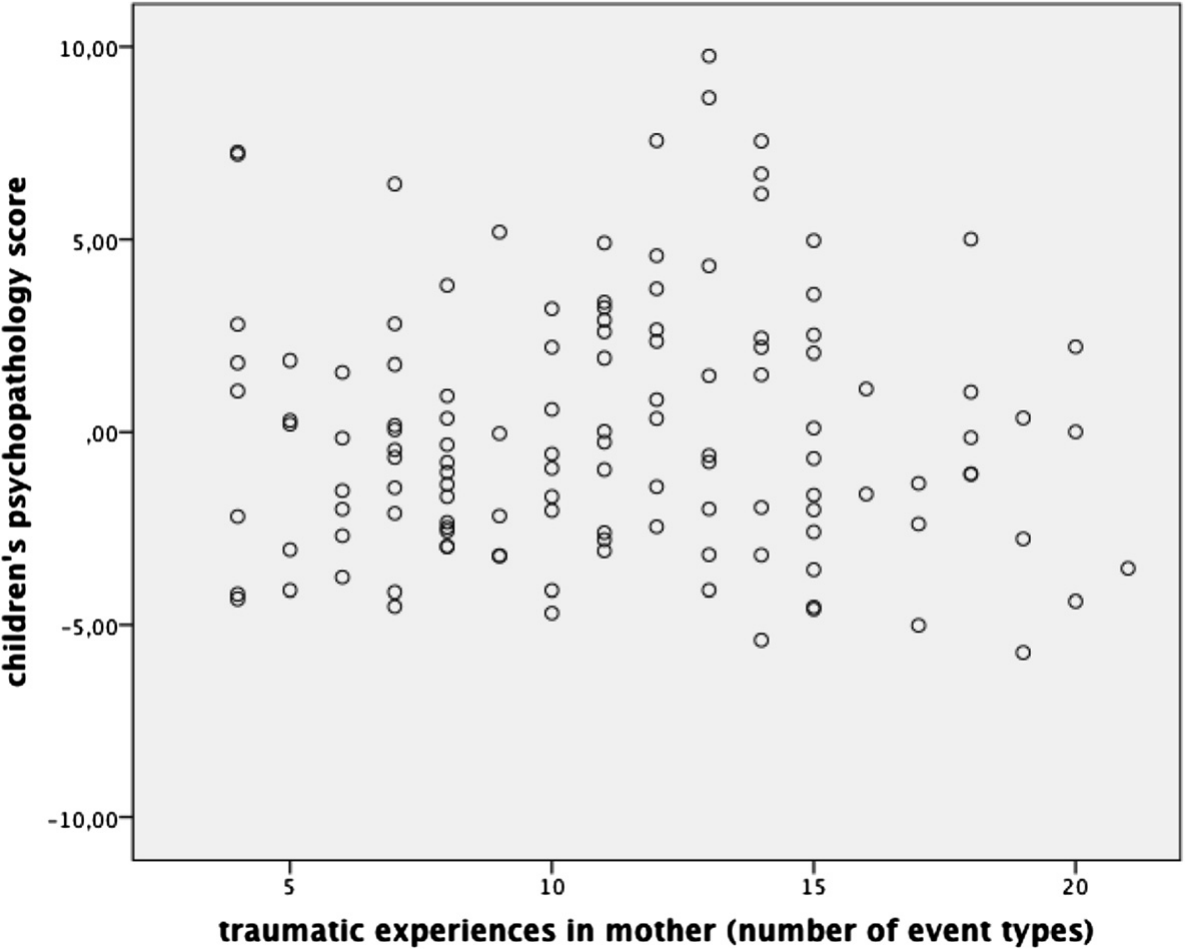
**Scatter plot and Pearson correlation coefficient for maternal traumatic experiences (number of event types) and children’s psychopathology score.** r = - .01, p = .94.

According to our first hypothesis, we examined Pearson correlation coefficient. Our results did not indicate an association between the number of traumatic events experienced by the mother and children‘s psychopathology (r = -.01, p = .94). See Figure 2 for a scatter plot. This result does not support our first hypothesis.

### Children’s psychopathology in association with maternal PTSD

**Figure 3:**
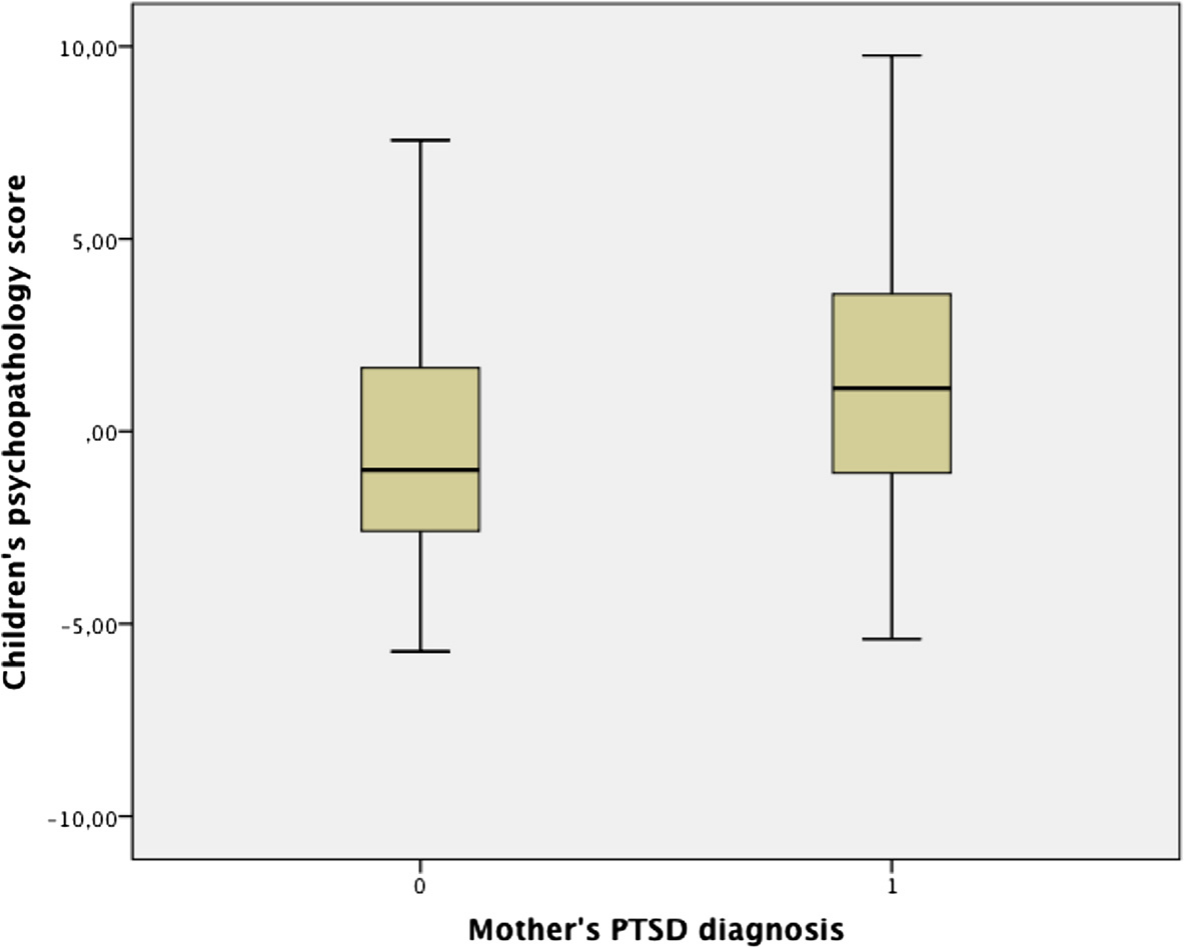
**Boxplot and t-values of t-Test for independent samples with maternal PTSD diagnosis as group variable and children’s psychopathology score as dependent variable.** t = - 1.61, p = .11.

Our second hypothesis represents the core hypothesis of our study. No group differences were identified in children’s psychopathology, comparing children of mothers with PTSD diagnosis and mothers without PTSD diagnosis (t = - 1.61, p = .11). See Figure 3 for a box plot. The result does not support our second hypothesis.

### Children’s psychopathology in association with maternal violence at home

**Figure 4:**
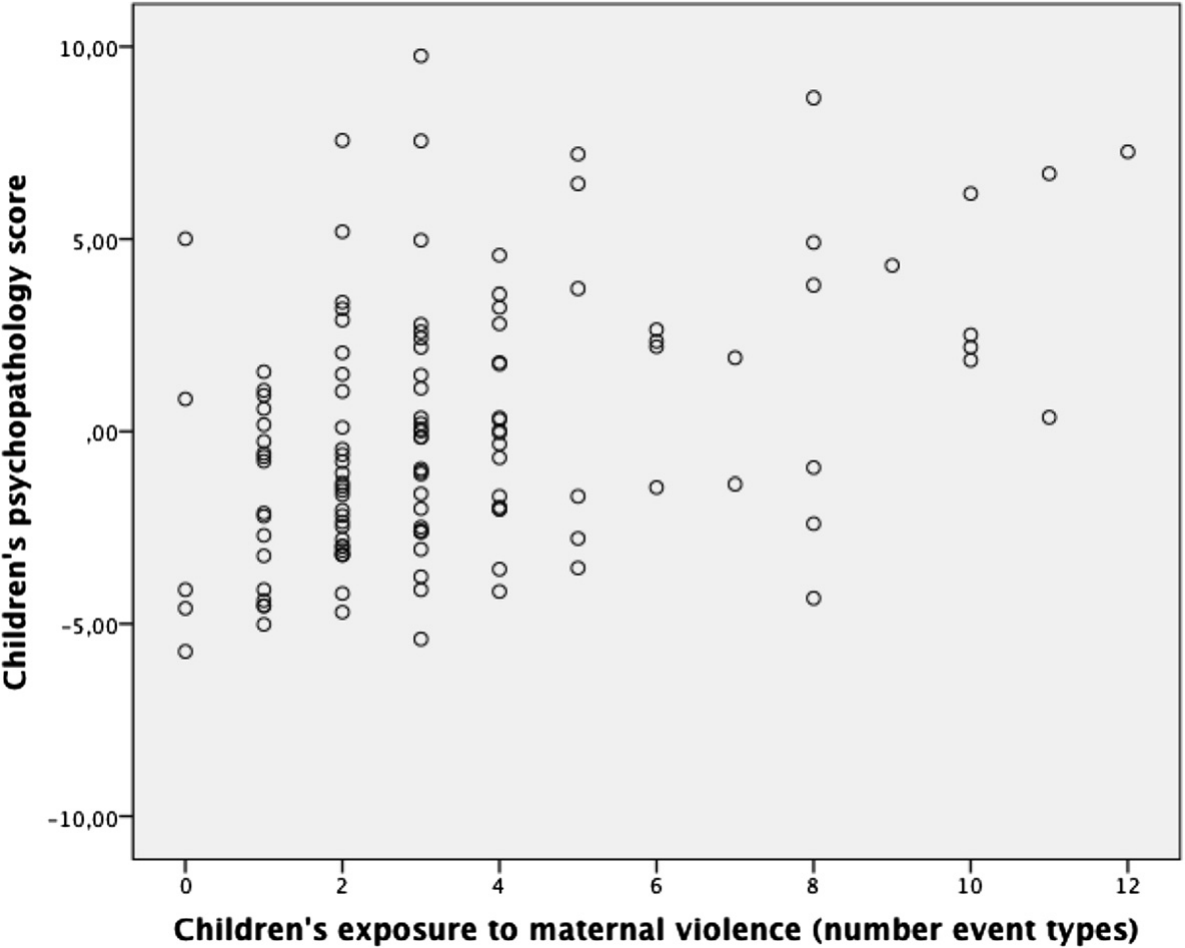
**Scatter plot and Pearson correlation coefficient for children’s exposure to maternal violence (number of event types) and children’s psychopathology score.** r = .59, p < .001.

According to our third hypothesis, we examined whether children’s experienced maternal violence at home is associated with children’s psychopathology. This was confirmed by a significant Pearson correlation of r = .59 (p < .001). Figure 4 shows a scatter plot for this association.

### Linear regression model with children’s psychopathology as dependent variable

In a hierarchical linear regression model we predicted in the first step that the children’s experienced maternal violence at home would explain a significant portion of the variance of the children’s psychopathology. With the addition of mother’s PTSD symptom score in the second step, the explained variance increased from 16% to 18% (F (2, 121) = 14.4, p < .001). Contrary to the amount of children’s experienced maternal violence at home (r = .41, p < .001) mother’s PTSD symptom (r = .18, p = .06) score did not achieve a significant contribution. Other confounding variables did not significantly explain any further variance in children’s psychopathology. For further details, see Table 1. These results support our third hypothesis and confute again our second hypothesis.

**Table 1:** Hierarchical regression model with children’s psychopathology score as dependent variable and children’s reported family violence conducted by the mother (event sum score) and maternal PTSD symptom score as independent variables

	**B**	**SE B**	**Beta**	**Zero-order correlation**	**p-value**
First step					
Constant	-1.84	0.46			< .001
Children’s experienced maternal violence at home (sum score of event types)	0.53	0.12	.41	.41	< .001
Second step					
Constant	- 2.44	.55			
Children’s experienced maternal violence at home (sum score of event types)	.52	.11	.40	.41	< .001
Mother’s PTSD symptom score	.05	.03	.16	.18	.06

Note: Full model’s adjusted R = .18; F (2,121) = 14.40, p < .001.

### Maternal violence at home in association with maternal PTSD

**Figure 5:**
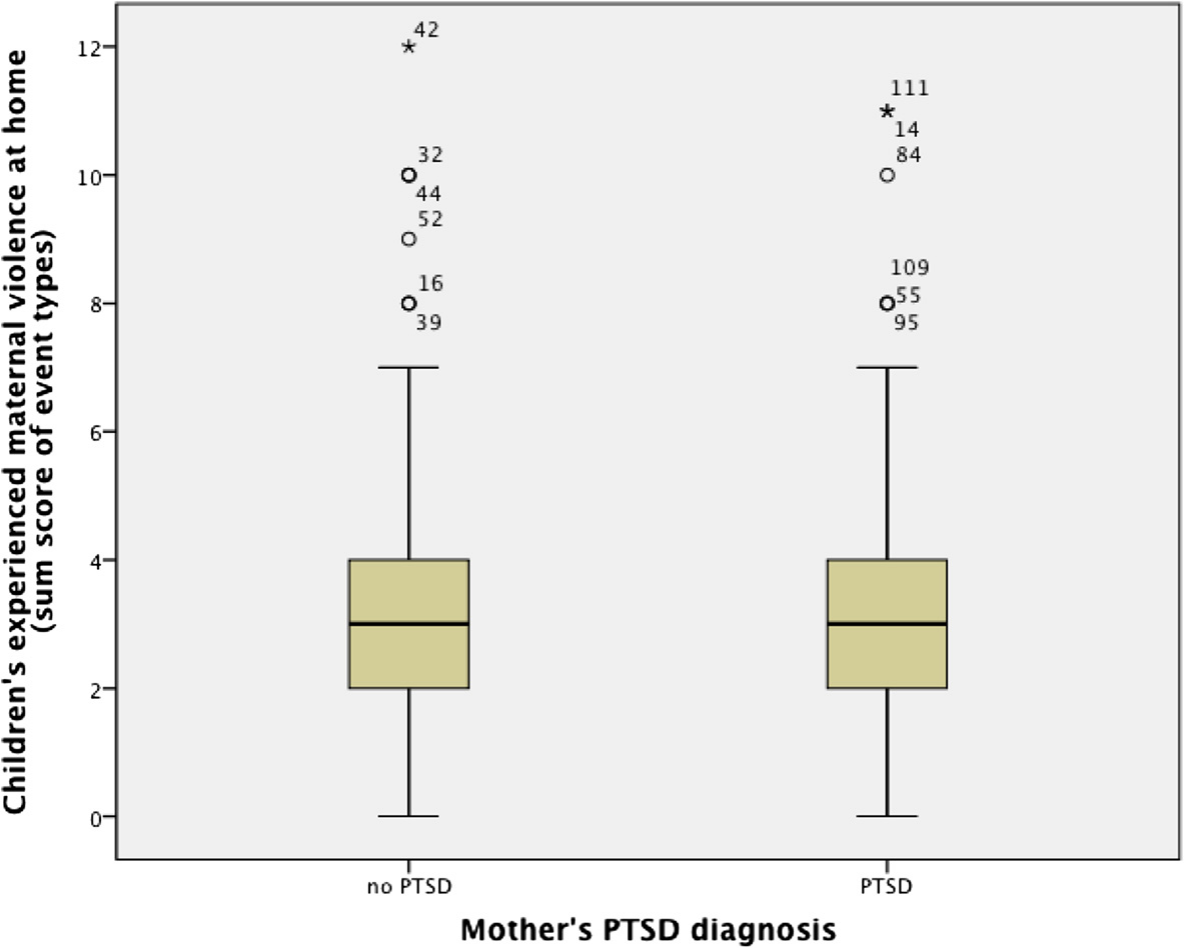
**Boxplot and t-values of t-Test for independent samples with maternal PTSD diagnosis as group variable and children’s experienced maternal violence at home (sum score of event types) as dependent variable.** t = - .73, p = .46.

No group differences were identified in children’s experienced maternal violence at home, comparing children of mothers with PTSD diagnosis and mothers without PTSD diagnosis (t = - .73, p = .46). This result confutes our fourth hypothesis. Figure 5 shows the respective box plot.

### Maternal violence at home in association with mothers own experiences of family violence during her childhood

**Figure 6:**
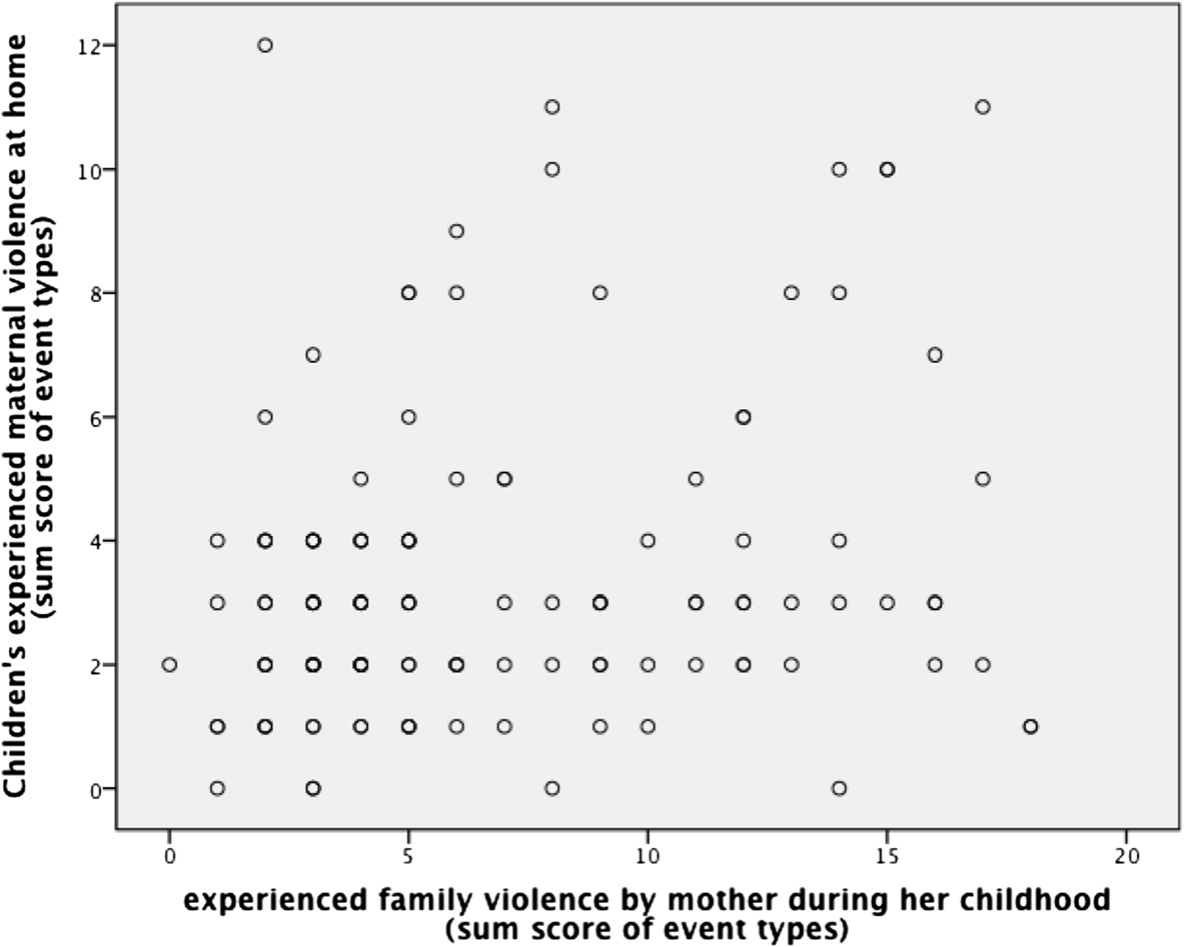
**Scatter plot and Pearson correlation coefficient for children’s experienced maternal violence at home (sum score of event types) and family violence experienced by the mother during her own childhood (sum score of event types).** r = .23, p < .01.

According to our fifth hypothesis, assuming an association between maternal used violence at home and experiences of family violence during her own childhood, we calculated a Pearson correlation coefficient and identified a significant association (r = .23, p < .01). Figure 6 shows the scatter plot. This result confirms our fifth hypothesis.

### Linear regression model with maternal violence at home as dependent variable

In order to understand how these two factors contribute simultaneously to the occurrence of children’s experiences of maternal violence at home, we calculated a hierarchical linear regression model using the maternal violence experienced by the children at home (sum score of event types) as dependent variable. In the first step we predicted that mother’s own exposure to family violence in her childhood (sum score of event types) explains a significant portion of the variance of maternal violence at home. With the addition of mother’s PTSD symptom score in the second step, 4% of the variance (F (2, 121) = 3.6, p < .05) could be explained. Other variables, including gender, number of siblings, educational level (current school grade) and family’s economical status did not explain any further variance in child psychopathology. For further details, see Table 2. These results contradict our forth hypothesis and support our fifth hypothesis.

**Table 2:** Hierarchical regression model with children’s experienced maternal violence at home (sum score of event types) as dependent variable and mother’s experienced family violence during her own childhood (sum score of event types) and maternal PTSD symptom score as independent variables

	**B**	**SE B**	**Beta**	**Zero-order correlation**	**p-value**
First step					
Constant	2.61	0.40			< .001
Mother’s experienced family violence during childhood (sum score of event types)	0.13	0.05	.23	.23	< .01
Second step					
Constant	2.69	.42			< .001
Mother’s experienced family violence during childhood (sum score of event types)	.14	.06	.27	.23	< .05
Mother’s PTSD symptom score	- .02	.03	- .07	.07	.51

Note: Full model’s adjusted R = .04; F (2,121) = 3.61, p < .05.

## Discussion

Our study did not identify an association between children’s psychopathology and maternal traumatic experiences and maternal PTSD. Instead, a significant relationship was found for children’s psychopathology and their experienced maternal violence at home. Furthermore, maternal violence against the child was related not only with child’s psychopathology but also with mother’s own experiences of family violence during their childhood.

The key finding of our study constitutes the missing association of children’s psychopathology with maternal PTSD. This result contradicts the common assumption of a direct *trans-generational trauma transmission*. Obviously, a large-scale catastrophe as the genocide is not necessarily specifically damaging the mental health of the children of survivors. This finding is supported by a number of review articles [45-47] and a comprehensive meta analysis on studies of children of Holocaust survivors, which states that the influence of the parents’ traumatic experiences on their children seems to be restricted to studies on clinical participants and cannot be viewed as a common phenomenon [48]. It has also been suggested that the behaviour of Holocaust survivor offspring may not be clinically distinctive but rather a specific personality configuration [47].

The second important finding of our study is that family violence affects the mental health and wellbeing of the children as opposed to maternal PTSD. Our study indicated an association of children’s mental health status and their experienced amount of maternal violence at home. Likewise, a number of studies identified psychological consequences of family violence for the children, such as anxiety [49,50], antisocial behaviour [21,51], aggression [52], and depression [51]. The explicit negative psychological consequences of family violence for a child may be explained not only by the experience of a frightening situation in an intimate familial environment but also by the absence of an adequate reaction from the parent and the lack of a trustful relationship. Abusive child-rearing practices do not provide a model for the child to develop adequate emotional regulation mechanisms and interpersonal relatedness, which in turn may lead to antisocial behaviour, such as aggression or Oppositional Deviant Disorder [53,54].

Furthermore, our study shows that children‘s experienced maternal violence is not associated with maternal PTSD symptom profiles, but with the amount of the mother‘s own experiences of family violence during her childhood. This sheds a new light on studies that reported that parental PTSD is associated with child rearing practices such as family violence [12-15]. It may not be the PTSD of the mother per se, but the family violence experienced by the mother, that increases the risk for both, the PTSD in the mother and the exposure to family violence in the child. This would support the notion of a *Cycle of Violence* and fit with results of a series of other studies [52,55-57]. The social learning process was assumed to represent the mechanisms behind this association [58]. Abusive parents represent a role model for abusive behaviour and the moral assurance that such behaviour is appropriate; furthermore, children growing up in an abusive household lack alternative non-violent conflict resolution strategies [59]. As family violence is mostly embedded in other family pathology [60], we assume additional risk factors, such as a violent social environment or lack of family support, which are not assessed in our study but which latently explain the impact of family violence on the children’s mental health. Furthermore, it is important to consider that child maltreatment cannot be separated from other disadvantaging factors, such as high everyday life stress or social isolation [60,61].

**Figure 7:**
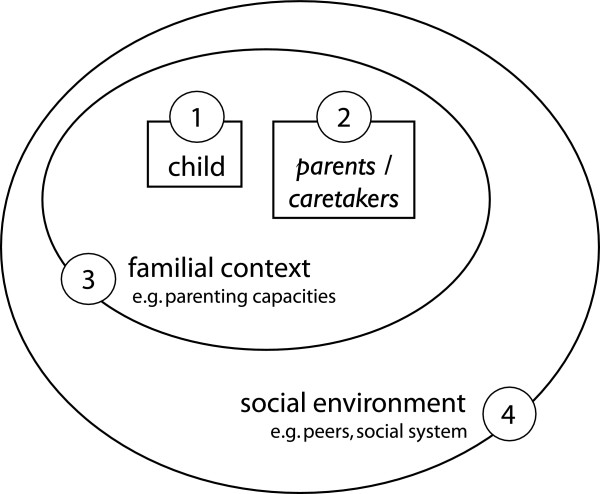
**Diagram illustrating the complexities of the investigation.** The child with all its strength and vulnerabilities should be the central focus of the investigation. Depending on various factors, e.g. genetic predispositions, children may be strengthened or may become more vulnerable by the knowledge of parental traumatic experiences [45]. Second, the mental health status of the child’s caretakers – in most cases the biological parents – must be considered. The parent itself may not only show PTSD in consequence of the experience of traumatic events, but develop also other pathological behaviour patterns, symptoms (e.g. complex traumatization, depression), or specific personality configurations. It must be considered that people exposed to traumatic events don’t only report negative consequences in their lives. Some show positive changes in their self-perception, in their perception of others and in the objective and meaning of their lives [62,63]. Third, the family context in general must be respected. It implies the impact of siblings or general family functioning, such as patterns of communication, parenting capacity, stress-coping strategies or general worldviews [45,46]. Fourth, the influence of the extra familial support system needs to be taken into account, such as peers, level of integration in the community, which may e.g. concern prejudice of minorities such as refugees or immigrants [45].

We conclude that the simple conclusion “a mother with PTSD has children with more symptoms of anxiety, depression and aggressive behaviour” is not valid. At the same time there might be symptoms and mechanisms that are not assessable with the methodology of quantitative measurements. Figure 7 illustrates the different levels that need to be considered when investigating *trans-generational trauma transmission*.

Some limitations warrant mention. As our data collection was cross-sectional, the cause and effect of relationships cannot be demonstrated. It is therefore possible that family violence was as much a cause as an effect of children’s symptoms. Furthermore, data about the father were not assessed, which might have explained additional variance of children’s symptoms.

The assessment of children’s psychopathology was also limited. Children’s anxiety symptoms were measured with the *HSCL-25*, an instrument designed for adults. Assessment of antisocial behaviour should use different sources of information, such as a parent or a teacher, providing different aspects of a child’s functioning [64,65]. As the amount of children’s stressful experiences might also represent an important predictor of children’s mental wellbeing, children’s traumatic experiences should have been assessed. Similarly, school and community violence may play an important role and should be assessed as well.

Still, this study explores the assumption of *transgenerational trauma transmission* and provides systematic information on two generations, assessed by local B.A. psychologists in a conflict region. All participants are from the same cultural background and allow a comparison of the children of traumatized and non-traumatized mothers. Furthermore, family violence was not just reported by the parents but the children, assuming less bias through social desirability.

The Rwandan women were highly motivated to participate in the study. In our experience, people in resource-poor regions are often highly motivated to make their voice “heard”. Usually most people want to contribute to a survey that may document daily living conditions and daily sufferings.

## Conclusions

Our findings do not support the common hypothesis of trans-generational consequences of parental PTSD, as we did not find evidence for an association between maternal PTSD and children’s mental health. Instead, experiences of maternal violence represented the most important predictor of children’s depression, anxiety, aggression and antisocial behaviour. In contrast with the theory on *trans-generational trauma transmission* children’s experience of maternal violence at home was not associated with maternal PTSD but with mothers own experience of family violence during her own childhood, a result that is in congruence with the hypothesis of the *Cycle of Violence*. We assume that there might be far more factors affecting child rearing practices and children’s mental wellbeing, factors which concern general life and not exclusively PTSD and traumatic past. Our results call for attention for the psychological consequences of family violence in African countries, where family violence is socially accepted as a fundamental child rearing practice. Additionally, our results confirm studies of other countries in the world investigating the *Cycle of Violence* and therefore support generalization of this hypothesis. The devastating consequences of family violence can be taught to parents, together with alternative child-rearing practices.

## Competing interests

Authors declare they have no conflict of interests.

## Authors’ contributions

MR carried out the realization and supervision of the study in Rwanda, performed statistical analysis and drafted the manuscript. FN and TE participated in planning design of the examination and supplemented the manuscript. All authors had full access to the data in the study. The first author takes responsibility for the integrity of the data and the accuracy of the data analysis. All authors read and approved the final manuscript.
